# Evaluating White Matter Lesion Segmentations with Refined Sørensen-Dice Analysis

**DOI:** 10.1038/s41598-020-64803-w

**Published:** 2020-05-19

**Authors:** Aaron Carass, Snehashis Roy, Adrian Gherman, Jacob C. Reinhold, Andrew Jesson, Tal Arbel, Oskar Maier, Heinz Handels, Mohsen Ghafoorian, Bram Platel, Ariel Birenbaum, Hayit Greenspan, Dzung L. Pham, Ciprian M. Crainiceanu, Peter A. Calabresi, Jerry L. Prince, William R. Gray Roncal, Russell T. Shinohara, Ipek Oguz

**Affiliations:** 1 Department of Electrical and Computer Engineering, The Johns Hopkins University, Baltimore, MD 21218 USA; 2 Department of Computer Science, The Johns Hopkins University, Baltimore, MD 21218 USA; 3 CNRM, The Henry M. Jackson Foundation for the Advancement of Military Medicine, Bethesda, MD 20817 USA; 4 Department of Biostatistics, The Johns Hopkins University, Baltimore, MD 21205 USA; 5 Centre For Intelligent Machines, McGill University, Montréal, QC H3A 0E9 Canada; 6 Institute of Medical Informatics, University of Lübeck, 23538 Lübeck, Germany; 7 Institute for Computing and Information Sciences, Radboud University, 6525 HP Nijmegen, Netherlands; 8 Diagnostic Image Analysis Group, Radboud University Medical Center, 6525 GA Nijmegen, Netherlands; 9 Department of Electrical Engineering, Tel-Aviv University, Tel-Aviv, 69978 Israel; 10 Department of Biomedical Engineering, Tel-Aviv University, Tel-Aviv, 69978 Israel; 11 Department of Neurology, The Johns Hopkins University School of Medicine, Baltimore, MD 21287 USA; 12 Penn Statistics in Imaging and Visualization Center, Department of Biostatistics & Epidemiology, University of Pennsylvania, Philadelphia, PA 19104 USA; 13 Center for Biomedical Image Computing and Analytics, Department of Radiology, University of Pennsylvania, Philadelphia, PA 19104 USA; 14 Department of Electrical Engineering and Computer Science, Vanderbilt University, Nashville, TN 37203 USA

**Keywords:** Magnetic resonance imaging, Multiple sclerosis, Biomedical engineering, Computational science, Software

## Abstract

The Sørensen-Dice index (SDI) is a widely used measure for evaluating medical image segmentation algorithms. It offers a standardized measure of segmentation accuracy which has proven useful. However, it offers diminishing insight when the number of objects is unknown, such as in white matter lesion segmentation of multiple sclerosis (MS) patients. We present a refinement for finer grained parsing of SDI results in situations where the number of objects is unknown. We explore these ideas with two case studies showing what can be learned from our two presented studies. Our first study explores an inter-rater comparison, showing that smaller lesions cannot be reliably identified. In our second case study, we demonstrate fusing multiple MS lesion segmentation algorithms based on the insights into the algorithms provided by our analysis to generate a segmentation that exhibits improved performance. This work demonstrates the wealth of information that can be learned from refined analysis of medical image segmentations.

## Introduction

Segmentation is one of the cornerstones of image processing; it is the process of automatic or semi-automatic detection of boundaries within an image. In a medical imaging context, segmentation is concerned with differentiating tissue classes (i.e., white matter vs. gray matter in the brain), identifying anatomy, or pathology. Motivating examples for the use of segmentation in medical imaging include content based image retrieval^1^, tumor delineation^2^, cell detection^3^ and motion tracking^4^, object measurement for size^5^, shape analysis^6^ evaluation, and myriad other uses^7–47^. The review articles by Pham *et al*.^48^ and Sharma *et al*.^49^ are a useful resource, providing an overview of the different applications of segmentation in medical imaging. A common feature of all this literature is the evaluation of the proposed segmentation algorithm either in comparison to previous work, or more importantly, to some manual/digital gold-standard. In fact, it is impossible to report new segmentation methods without such evaluation; it therefore follows that evaluating medical image segmentation algorithms is important.

There have been many methods employed in the evaluation of medical image segmentation algorithms. Voxel-based methods such as intra-class correlation coefficient (ICC)^50,51^, Sørensen-Dice Index^52,53^, and Jaccard coefficient^54^ can provide insight about the agreement between a ground truth segmentation and an algorithm or between two segmentations to establish their similarity. Related measures include Cohen’s kappa^55^, detection and outline error estimates (DOEE)^56^, as well as true/false positives and their corresponding negatives. Distance metrics, such as symmetric surface distance, can directly show how far a segmentation deviates from a desired object boundary or boundary landmarks^57^. See Taha and Hanbury^58^ for a more detailed review and comparison of evaluation approaches for medical image segmentation.

An issue with these traditional measures for medical image segmentation is the focus on reporting a global measure. For simple object detection tasks, where a single object is under consideration, these global scores directly relate to the segmentation peformance of the single object under consideration. However, in the case of an unknown number of objects, such as vertebrae detection in the spine, these evaluations may be masking underlying issues. Thus, considering the number of correctly detected objects is an important measure of the accuracy of such algorithms. This is exacerbated in cases were the number of objects is not known *a priori*, such as in cell segmentation. Ideally, we would like to evaluate these segmentations on an object by object basis, which might be perfectly fine in the case of spinal vertebrae or lung lobes. However, the evaluation of hundreds (or even thousands) of objects on an object-by-object basis is impractical due to the large number of cases.

A prime example of an application domain with a variable number of objects is multiple sclerosis (MS) lesion segmentation from magnetic resonance image (MRI) scans of the brain or spine. White matter lesions (WMLs) are a hallmark of MS and their segmentation and quantification are critical for clinical purposes and other applications^59–62^. Many approaches to MS lesion segmentation have been proposed: artificial^63^ and convolutional neural networks^64^; Bayesian models^65^; Gaussian mixture models^66^; graph cuts^67^; random forests^68^; and many others^36,38,68–110^. Review articles by Lladó *et al*.^111^ and García-Lorenzo *et al*.^112^ provide context and an historical insight into the field. Research is continuing in this area with new methods being developed at an almost breakneck pace, with several grand challenges (MICCAI 2008^113^, ISBI 2015^114,115^, MICCAI 2013^116^, MICCAI 2015^117^, MICCAI 2016^118^, MICCAI 2018^119^) being organized to help establish the state-of-the-art. With recent work having focused on standardizing these grand challenges^120^ to improve the interpretability and stability of results. However, the evaluation of new algorithms continues to rely heavily on Dice and Jaccard overlaps, lesion counts, and total lesion volume (known as lesion load). This, as noted above, limits our ability to fully assess the detailed characteristics of an algorithm, and in particular to differentiate their performance. Moreover, reliance on these measures impedes our ability to refine and improve existing algorithms.

In this work, we build on our previous work^121^ and address these concerns by illustrating potentially useful information that can be obtained from a deeper understanding of the SDI. In Section 2, we provide an historical review of the SDI and related work. In Section 3, we present the methods used in this work, describe our data, and review some state-of-the-art WML segmentation algorithms that we will use for comparison purposes. In Section 4, we use the SDI to understand the differences between two raters (an inter-rater comparison), we apply similar analyses to compare four state-of-the-art algorithms. We demonstrate a naive hybrid algorithm based on cross-validation and our SDI analysis, in Section 5. To avoid confusion, we point out that our evaluations are focused on binary segmentations of WMLs, though our approach can apply to any binary segmentation where the number of objects is not known *a priori*, such as cell segmentation or star detection^122^.

## Background

Lee R. Dice, wishing to understand the association between species, proposed what he called the *Coincidence Index* in his 1945 paper^52^ as a statistic to gauge the similarity of two samples. The measure was introduced to address shortcomings in the work of Forbes^123^, who had suggested using a *coefficient of association* to address the problem. The work of Forbes resulted in a near binarized measure between species association negating its usefulness. Both measures have values in the range $\mathrm{[0,}\,\mathrm{1]}$, however, in contrast to the coefficient of association, the Coincidence Index could use the full extents of this range in a meaningful manner. Independent of Dice, Thorvald Sørensen introduced an almost identical measure^53^, the difference being that Sørensen developed a formulation focused on the absence of species rather than their presence. We will refer to this measure as the Sørensen-Dice index (SDI), noting that it has been known by many names: Dice’s coefficient, Dice overlap, Sørensen index, etc. However several other, less obvious, names have appeared in the literature. For example, one of the early papers applying the measure to medical imaging was Zijdenbos *et al*.^124^, which resulted in the measure being referred to as the Zijdenbos similarity index by some authors in the intervening years^43,125–129^.

The formulation for the SDI, which we provide below, is for the case of exploring two co-occurring species (or in our instance elements). However, multi-element extensions have been reported; known generally as the Bray-Curtis similarity^130^ though also known as Pielou’s percentage similarity^131^ or the quantitative Sørensen index, and also the Generalized Dice Coefficient^132^. We will restrict this work to the case of two co-occurring elements. The SDI is closely associated with the Jaccard index^54^, it is trivial to convert the scores of one to the other. We have focused this work on the SDI; however, we note that analyses similar to those presented in Section 4 can readily be performed using the Jaccard index. Now, for sets $A$ and $B$ we define the SDI as,

$${\rm{SDI}}(A,B)=\frac{2|A\cap B|}{|A|+|B|},$$

where |·| denotes cardinality. We note that the SDI is a pseudo-semimetric; the SDI does not satisfy the Identity Property (pseudo) or the Triangle Property (semi-) of a metric.

Zijdenbos *et al*.^124^ in introducing SDI to medical imaging—by deriving the SDI formulation from the Kappa coefficient essentially mirroring the work of Dice five decades earlier (as Zijdenbos *et al*. duly note)—was providing a way to standardize comparisons between different WML segmentation algorithms. The work has clearly had a profound effect on the field, with the original paper of Zijdenbos *et al*. having well over 1,000 citations. The SDI (under its many monikers) has now become a standard way for validating the improvement of segmentation algorithms. Dice when introducing the SDI in 1945, simultaneously presented the computation of a ${\chi }^{2}$-test for the measure immediately showing “*whether the combinations of species in the samples* … *may possibly be due to chance errors in random sampling*.”^52^. This is a powerful aspect of Dice’s work. However, the original presentation and the Kappa coefficient formulation highlight the issue with SDI, namely, it is designed for objects that have some interpretable correspondence. In the case of liver segmentation, for example, there is one object under consideration and a one-to-one correspondence between objects when comparing two liver segmentations and the use of SDI makes sense. Thus there is an implicit assumption that the number of segmentation targets is known *a priori*, e.g., whether it is one liver, two hippocampi, or five lung lobes. There is, however, an entire class of segmentation problems where the number of objects is unknown. The problem of WML segmentation is a good example, where the number of objects can vary from zero to hundreds. In particular for WML segmentation there can be disagreement even between radiologists about the exact number of lesions present in a particular region. In such cases, the problem of object detection and segmentation are conflated and performance evaluation should make some effort to distinguish this aspect accordingly. Unfortunately, it has been customary for the image-wide SDI to be used to reflect both aspects of this problem and this leads to an oversimplification in trying to distinguish the characteristics of various algorithms. Alternatively, considering object detection by counting the number of detected objects as a measure of success/failure is also misleading, as object counts include both false positives and false negatives; moreover an object count misrepresents large objects that have been split into multiple smaller objects and vice versa. Another concern with the SDI is its inability to incorporate the size of objects within its score. This is of great importance with WML segmentation; a segmentation algorithm that misses small lesions may be of less clinical use as new (necessarily small) lesions can be indicative of disease progression, this is not reflected in the global SDI score.

**Figure 1:**
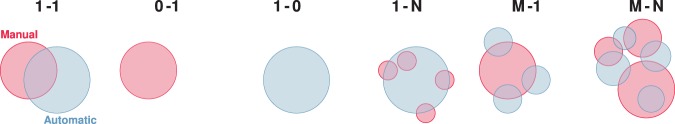
Illustrated from left to right are examples of the six classes for the Nascimento nomenclature; the leftmost panel is the case of “Correct Detection” also known as 1-1 correspondence between the manual segmentation and the automated segmentation. The next two cases are “Detection Failure” or 0-1 correspondence, were there is a manual segmentation but no overlapping object in the automated segmentation, and “False Alarm” or 1-0 correspondence. The next three cases are the object detection classes known as “Merge”, “Split”, and “Split-Merge”. The 1-N or “Merge” case occurs when the automated segmentation has merged the multiple objects from the manual segmentation into a single object. Next is M-1, “Split”, in which a single manually segmented object has been split into multiple objects by the automated approach. Finally, on the right, M-N, are multiple manually segmented objects split and merged by the automated segmentation.

We can address some of these concerns by introducing the segmentation classification for known object correspondences developed by Nascimento and Marques^133^. In their work, a nomenclature was described to classify the various types of matches that can occur between two segmentations. Given two segmentations, one ground truth and the other the output of an automated algorithm, we can think of the connected components of these two segmentations; moreover, we can consider how these connected components relate to each other. Specifically for WMLs, if the manual segmentation has identified a lesion, then we can ask if the automated segmentation has identified a single corresponding connected component that overlaps with the manual segmentation but may not have the same extents. In such a case, there is said to be a 1-1 match between the two segmentations, and we can think of this lesion as being correctly detected. Hence, we can think of all the lesions that have been correctly detected, and consider the SDI—or any evaluation measurement for that matter—of the class of correctly detected lesions. We adopt the Nascimento nomenclature and refer to this class as “Correct Detection”. Using this approach we can readily define two other classes that arise when comparing manual and automatic segmentations. The first class is “False Alarm” which characterizes lesions that the algorithm identifies and the manual segmentation does not; the second class is “Detection Failure” which is defined as lesions in the manual segmentation that the algorithm fails to identify. These first three classes are illustrated in Fig. 1, as the three left most panels. We note that these three classes have been used in the past to distinguish segmentations; however, they were used on a global, per voxel, basis. We will use them on a per lesion basis.

There are three additional classes that are now required to complete the taxonomy for classifying the agreement between the manual and algorithm segmentations. First, consider the case where the manual delineation has identified several small lesions close together, whereas the algorithm has identified this cluster of lesions as a single lesion (see Fig. 1 for an example). The algorithm has not failed to detect the lesions but has, however, failed to disambiguate the lesions. We can think of the algorithm (for classification purposes) as having merged the lesions, which is why we refer to this as the “Merge” class. Upon identifying the merge class, it becomes self-evident that there must be a reciprocal class in which the algorithm has subdivided a single manually-identified lesion; we refer to this as the “Split” class. Finally, we identify a “Split-Merge” class in which, for example, the algorithm has identified four lesions that overlap with three lesions in the manual segmentation. Both segmentations agree there are lesions, but the boundaries between the confluent lesions are in disagreement. These six different classes of object agreement originate from the work of Nascimento and Marques^133^. We observe that these six classifications represent a complete taxonomy for all possible overlap combinations between two segmentations. Illustrations for the merge, split, and split-merge classes are shown in the three right most panels of Fig. 1.

In this work, we take these ideas of incorporating object detection classification and explore their potential application in medical image segmentation. This is important because the global SDI, or any global metric, can obscure performance in some classes of objects. However, global SDI can be a sufficient and defining statistic in the case of a fixed number of detectable objects. We specifically apply the detection classification idea to the SDI computed on WML segmentations to enhance the automated segmentations and improve our process for creating manual segmentations.

## Methods and Materials

### Classifying segmentation overlap

Assume that we have two binary segmentations, ${\mathbb{U}}$ and ${\mathbb{V}}$, of an image, which are both trying to identify particular objects in which the exact quantity of objects is unknown *a priori*. We first identify the 6-connected components in 3D (4-connected components in 2D) in each segmentation, and denote these objects as ${u}_{i}$ and ${v}_{j}$ coming from ${\mathbb{U}}$ and ${\mathbb{V}}$, respectively. Then for each object ${u}_{i}$, we identify corresponding objects in ${\mathbb{V}}$ as any ${v}_{j}$ which has a non-empty intersection with ${u}_{i}$. We denote the set of such corresponding objects by $C({u}_{i})=\{{v}_{j}|{v}_{j}\in {\mathbb{V}},{u}_{i}\cap {v}_{j}\ne \varnothing \}$ and we similarly denote the set of corresponding objects of ${v}_{j}$ in ${\mathbb{U}}$ as $C({v}_{j})=\{{u}_{i}|{u}_{i}\in {\mathbb{U}},{u}_{i}\cap {v}_{j}\ne \varnothing \}$. We observe that the cardinality of $C({u}_{i})$ determines the nature of the object detection classification; for example, if $|C({u}_{i})|=1$ and if for ${v}_{j}\in C({u}_{i})$ we have $|C({v}_{j})|=1$ then the objects are in a 1-1 correspondence which would equate to the “Correct Detection” class as used by Nascimento and Marques^133^. If, however, $|C({u}_{i})|={\rm{M}}$ (where $1 < {\rm{M}}$) and for each ${v}_{j}\in C({u}_{i})$ we have $|C({v}_{j})|=1$, then the objects are in an M-1 correspondence, which would be the “Split” class.

Thus far, the exact nature of ${\mathbb{U}}$ and ${\mathbb{V}}$ has not been stated, though it has been implied that they correspond to a manual segmentation and an automated segmentation, which is definitely a useful and common case. However, we want to expand this idea to include the case where both ${\mathbb{U}}$ and ${\mathbb{V}}$ are manual segmentations. This allows us to offer insight about the behavior of manual raters. For example, if the number of objects that are being merged, split, and split-merged between two human raters is high then it may reflect disagreements about interpreting object boundaries; it may also reflect the noise level in the images. If the number of objects in these same categories varies from low to high across a cohort, it may reflect inconsistent rater behavior or dissonant data. Thus, we can make observations about the inter-rater behavior of these raters on specific data. Moreover, if the two manual segmentations come from the same rater, we can explore intra-rater consistency. We have expanded the nomenclature of Nascimento and Marques to cover the case of comparing two manual segmentations (see Table 1). Another possible scenario, although not studied here, is the comparison of segmentations over time. The match type listed in Table 1 is readily computed as the cardinality of the appropriate $C({u}_{i})$ − $C({v}_{j})$ pairs.

**Table 1:** Our updated nomenclature, expanding on the work of Nascimento and Marques^133^ which focused on the comparison between manual and automated segmentations (Algorithm vs. Manual), to also cover the case when two manual segmentations are being compared (Manual vs. Manual).

Match Type	Manual vs. Manual	Algorithm vs. Manual
**1-1**	Expert Agreement	Correct Detection
**1-N**	Ambiguous Masks	Merge
**M-1**	Ambiguous Masks	Split
**M-N**	Ambiguous Masks	Split-Merge
**0-1**	Expert Disagreement	Detection Failure
**1-0**	Expert Disagreement	False Alarm

Examples of the different classes, for the situation of Algorithm vs. Manual, are shown in Fig. 1. The top four classes (1-1, 1-N, M-1, and M-N) represent cases of segmentation agreement, though the number of lesions and the boundary of those lesions is disputed. Whereas the bottom two classes (1-0, 0-1) are the classes which summarize segmentation disagreement.

In addition to our extensions of the Nascimento nomenclature^133^, we choose to plot the volume of each individual object, specifically WMLs, against the SDI. In doing so, we avoid the inherent volume insensitivity of the SDI by presenting both the volume and SDI of each individual object.

### Data

The data consists of MR images divided into two cohorts: (1) Training data set; and (2) Test data set. The Training data set consists of five subjects, four with four time-points and one subject with five time-points. The Test data set includes fourteen subjects, ten subjects with four time-points, three subjects had five time-points, and the final subject had six time-points. Two consecutive time-points are separated by approximately one year for all subjects. Table 2 includes a demographic breakdown for the training and test data sets. The data are available for download from the 2015 Longitudinal Lesion Segmentation Challenge Website (http://smart-stats-tools.org/lesion-challenge).

**Table 2:** Demographic details for both the training and test data set.

Data Set	N (M/F)	Time-Points Mean (SD)	Age Mean (SD)	Follow-Up Mean (SD)
Training	5 (1/4)	4.4 (±0.55)	43.5 (±10.3)	1.0 (±0.13)
RR	4 (1/3)	4.5 (±0.50)	40.0 (±07.6)	1.0 (±0.14)
PP	1 (0/1)	4.0 (±0.00)	57.9 (±0.00)	1.0 (±0.04)
Test	14 (3/11)	4.4 (±0.63)	39.3 (±08.9)	1.0 (±0.23)
RR	12 (3/9)	4.4 (±0.67)	39.2 (±09.6)	1.0 (±0.25)
PP	1 (0/1)	4.0 (±0.00)	39.0 (±0.00)	1.0 (±0.04)
SP	1 (0/1)	4.0 (±0.00)	41.7 (±0.00)	1.0 (±0.05)

The top row is the information of the entire data set, while subsequent rows within a section are specific to the patient diagnoses. The codes are RR for relapsing remitting MS, PP for primary progressive MS, and SP for secondary progressive MS. N (M/F) denotes the number of patients and the male/female ratio, respectively. Time-points is the mean (and standard deviation) of the number of time-points provided to participants. Age is the mean age (and standard deviation), in years, at baseline. Follow-up is the mean (and standard deviation), in years, of the time between follow-up scans.

Each scan was imaged and preprocessed in the same manner, with data acquired on a 3.0 Tesla MRI scanner (Philips Medical Systems, Best, The Netherlands) using the following sequences: a ${t}_{1}$-weighted (${T}_{1}$-w) magnetization prepared rapid gradient echo (MPRAGE) with TR = 10.3 ms, TE = 6 ms, flip angle = 8°, and $0.82\times 0.82\times 1.17$ mm^3^ voxel size; a double spin echo (DSE) which produces the PD-w and ${t}_{2}$-w images with TR = 4177 ms, TE_1_ = 12.31 ms, TE_2_ = 80 ms, and $0.82\times 0.82\times 2.2$ mm^3^ voxel size; and a ${T}_{2}$-w fluid attenuated inversion recovery (FLAIR) with TI = 835 ms, TE = 68 ms, and $0.82\times 0.82\times 2.2$ mm^3^ voxel size. The imaging protocols were approved by the local institutional review board.

**Figure 2:**
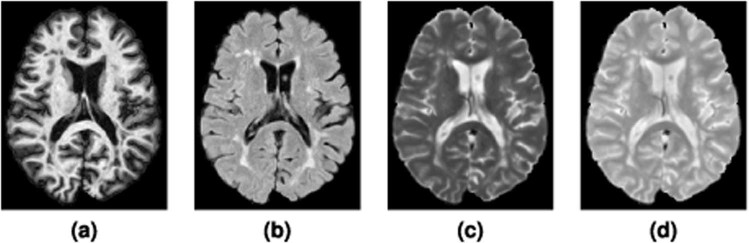
Shown are the **(a)** MPRAGE, **(b)** FLAIR, **(c)** ${T}_{2}$-w, and **(d)** PD-w images for a single time-point from one of the provided Training data set subjects after the preprocessing described in Section 3.2.

Each subject underwent the following preprocessing steps: the baseline (first time-point) MPRAGE was inhomogeneity-corrected using N4^134^, skull-stripped^135^, dura stripped^61^, followed by a second N4 inhomogeneity correction, and rigid registration to a 1 mm^3^ isotropic MNI template. We have found that running N4 a second time after skull and dura removal is more effective than a single application at reducing inhomogeneity in the images (see Fig. 2 for an example training image after preprocessing). Once the baseline MPRAGE is in MNI space, it is used as a target for the remaining images. The remaining images include the baseline ${T}_{2}$-w, PD-w, and FLAIR, as well as the scans from each of the follow-up time-points. These images are N4 corrected and then rigidly registered to the 1 mm isotropic baseline MPRAGE in MNI space. The skull and dura stripped mask from the baseline MPRAGE are applied to all the subsequent images, which are then N4 corrected again. The preprocessing steps were performed using JIST (Version 3.2)^136^.

All the images in the Training and Test data sets had their lesions manually delineated by two raters in the MNI space. Rater #1 had four years of experience delineating lesions at the time, while Rater #2 had 10 years experience with manual lesion segmentation and 17 years experience in structural MRI analysis at that time. We note that the raters were blinded to the temporal ordering of the data. The protocol for the manual delineation followed by both raters is provided in Carass *et al*.^115^.

### Consensus delineation

**Figure 3:**
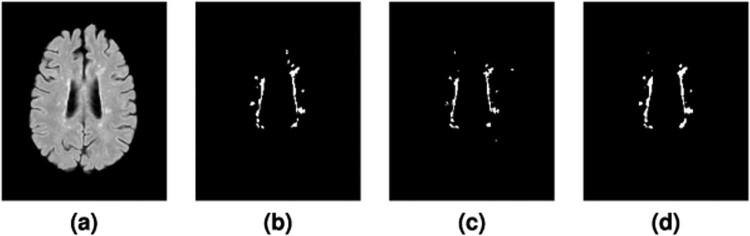
Shown is an axial slice of the **(a)** FLAIR for a single time-point from one of the Test data set subjects, and the corresponding mask by **(b)** Rater #1, **(c)** Rater #2, and the **(d)** Consensus Delineation.

We construct a Consensus Delineation for each test data set by using the simultaneous truth and performance level estimation (STAPLE) algorithm^44^. The Consensus Delineation uses the two manual delineations created by our raters as well as the output from all fourteen algorithms that submitted to the 2015 Longitudinal Lesion Segmentation Challenge^114,115^. The manual delineations and the fourteen algorithm delineations are treated equally within the STAPLE framework. Figure 3 shows an example slice from our Test data set with the corresponding delineations from Rater #1, #2, and the Consensus Delineation.

### Comparison methods

In Section 4.2, we explore what can be learned from the top four methods included in the 2015 Longitudinal Lesion Segmentation Challenge^114,115^; those methods are outlined below.

 **DIAG**

*Convolution Neural Networks for MS Lesion Segmentation*

(M. Ghafoorian and B. Platel)

The DIAG utilizes a convolutional neural network (CNN) with five layers in a sliding window fashion to create a voxel-based classifier^137^. As input the CNN used all the available modalities, with each modality contributing an image patch of size $32\times 32$.

 **IMI**

*MS-Lesion Segmentation in MRI with Random Forests*

(O. Maier and H. Handels)

The IMI method trained a random forest (RF) with supervised learning to infer the classification function underlying the training data^91^. The classification of brain lesions in MRI is a complex task with high levels of noise, hence a total of 200 trees are trained without any growth-restriction.

 **MV-CNN**

*Multi-View Convolutional Neural Networks*

(A. Birenbaum and H. Greenspan)

MV-CNN is a method based on a Longitudinal Multi-View CNN^138^. The classifier is modeled as a CNN, whose input for every evaluated voxel are patches from axial, coronal, and sagittal views of the available modalities^64^.

 **PVG One**

*Hierarchical MRF and Random Forest Segmentation of MS Lesions and Healthy Tissues in Brain MRI*

(A. Jesson and T. Arbel)

The PVG method built a hierarchical framework for the segmentation of a variety of healthy tissues and lesions. At the voxel level, lesion and tissue labels are estimated through a MRF segmentation framework that leverages spatial prior probabilities for nine healthy tissues through multi-atlas label fusion (MALF). A RF classifier then provides region level lesion refinement.

**Figure 4:**
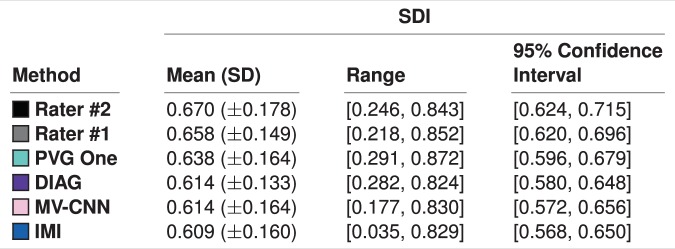
Mean, standard deviation (SD), and range of the SDI against the Consensus Delineation for the two human raters and the top four algorithms (as ranked by their SDI). We also include the 95% confidence interval of the mean SDI.

We note that the selected four algorithms had the highest ranked SDI amongst the fourteen algorithms against the Consensus Delineation, as shown in Fig. 4, which also reports the SDI for our raters against the Consensus Delineation. We note that the mean SDIs reported in Fig. 4 are all within one standard deviation of each other and well within their pairwise standard error. Using the global measure of mean SDI would suggest very little difference in the behavior of these four algorithms; however, as we will see in Section 4.2 this is not the case. We also report the 95% confidence intervals of the mean SDIs for the manual raters and the four methods in Fig. 4.

## Case Studies

We explore two case studies: (1) an inter-rater comparison across the Test data set; and (2) an exploration of four algorithms used in the 2015 Longitudinal Lesion Segmentation Challenge, on the same Test data set.

### Inter-rater comparison

**Figure 5:**
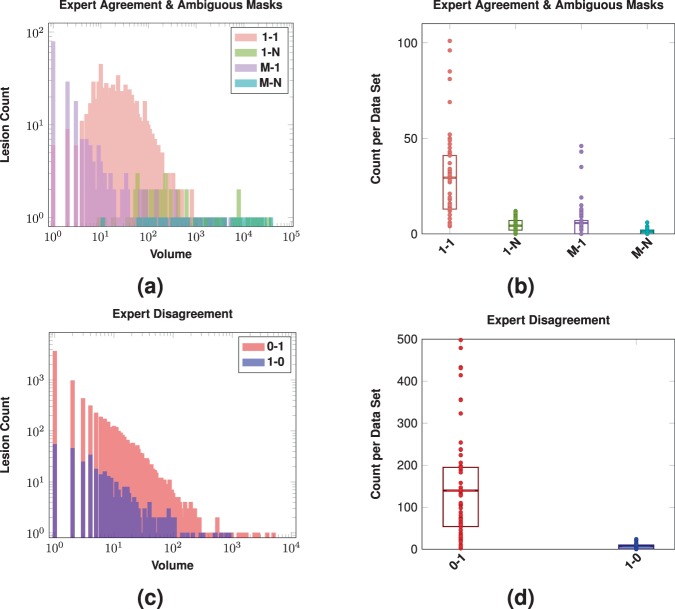
Shown in **(a)** are log-scale histograms depicting the Expert Agreement and Ambiguous Masks for our inter-rater comparison. The histograms show the volume ($x$-axis) and the count of lesions ($y$-axis) of that size. The volume of the lesions is the volume assigned by Rater #2. The Expert Agreement case (1-1) shows those lesions that had a one-to-one correspondence between lesions identified by Rater #1 and #2. The Ambiguous Masks classes (1-N, M-1, and M-N) are also shown. Shown in **(b)** are the counts on a per data set basis for the four different Expert Agreement and Ambiguous Masks cases; a dot denotes the respective count for one of the 61 test data sets, the rectangles represent the inter quartile range (IQR), and the horizontal bars are the means. Shown in **(c)** are log-scale histograms depicting the two Expert Disagreement cases for our inter-rater comparison. The histograms show the volume ($x$-axis) and the count of lesions ($y$-axis) of that size that were identified by Rater #1 but not Rater #2 (1-0) or identified by Rater #2 but not Rater #1 (0-1). The volumes come from the rater that identified the lesion. Shown in **(d)** are the counts on a per data set basis for the two different Expert Disagreement cases; a dot denotes the respective count for one of the 61 test data sets, the rectangles represent the IQR, and the horizontal bars are the means.

**Figure 6:**
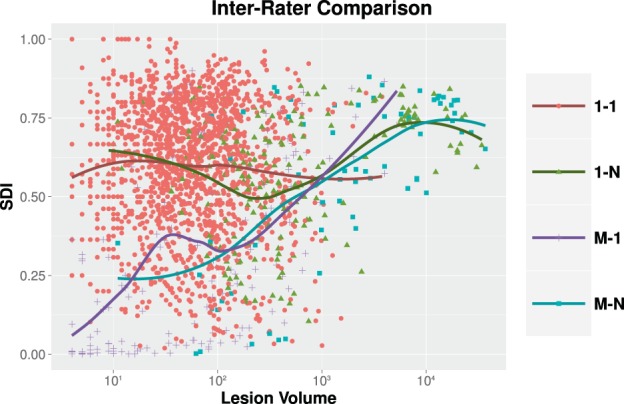
For our inter-rater comparison, we show per-lesion SDI for the expert agreement cases as a function of the lesion volume (color coded by lesion classification). The volume of the lesions is the volume assigned by Rater #2. For each category, the dots are individual lesions and the solid lines are a LOESS best fit^139,140^.

Shown in Fig. 5 are representations of the inter-rater detection classes, see Table 1 for details. We also present the Expert Agreement and Ambiguous Masks classes in Fig. 6, where we show the per-lesion SDI trends for these classes. The expert disagreement cases are those cases in which one rater has identified a lesion and the other rater has no lesion which overlaps the identified lesion.

Figure 5(a) shows histograms depicting the Expert Agreement and Ambiguous Masks cases for our inter-rater comparison. The histograms show the volume versus the count of lesions of that particular size. We see from Fig. 5(a), that a large number of small lesions identified by Rater #2 split single lesions identified by Rater #1, see the split ( M-1) case. Additionally, we see that while the split-merge class has a broad range (minimum size 11 mm^3^ upto a maximum size of 36,967 mm^3^) it has a count of one whenever such split-merge cases occur, suggesting that such cases are rare. Figure 5(b) shows the number of cases of each class on a per data set basis. We note that Fig. 5(b) echos the observations from Fig. 5(a) that the split-merge class has a very low incidence rate. We also note that the number of cases in the object detection agreement class is considerably higher than either of the other three classes.

Figure 5(c) shows histograms depicting the two Expert Disagreement cases for our inter-rater comparison. The histograms show the volume and the count of lesions of that particular size that were identified by one rater but not by the other rater. Figure 5(d) shows the counts on a per data set basis for the two different Expert Disagreement cases; a dot denotes the respective count for one of the 61 test data sets. From Fig. 5(c,d), we have that Rater #1 identified 388 lesions that Rater #2 did not, where 14.18% were of size 1 mm^3^ (equivalently one voxel) and 61.34% were of size 10 mm^3^ or less. The total of 388 unidentified lesions can also be computed as the sum of the 1-0 category in Fig. 5(d). Conversely, Rater #2 identified 8,514 lesions that Rater #1 did not, 43.35% of which had a size of 1 mm^3^ and 75.06% had a size of 10 mm^3^ or less. The total of 8,514 unidentified lesions can also be computed as the sum of the 0–1 category in Fig. 5(d). We note that the identification of small lesions by Rater #2 shown in Fig. 5(c) would appear consistent with our observations from Fig. 5(a) that lesions identified by Rater #1 are identified as groups of smaller lesions by Rater #2. Clearly, it is difficult for raters to agree on small lesions; however, our two raters failed to agree on 8,902 lesions meaning that there was not another (larger or smaller) lesion with any overlap for these lesions. Given that the total number of uniquely identified lesions was 11,245 then the 8,902 represents 79.2% of the total number of identified lesions.

Our first key observation is the large number of 1 mm^3^ lesion detection failures may point to our choice of connectivity model, which is related to Rater #2’s interpretation of connectivity. Had we used an 18-connectivity model, the total number of 1 mm^3^ lesions would have been 21 for Rater #1 and 1,163 for Rater #2, as opposed to 79 for Rater #1 and 3,837 for Rater #2 when using 6-connectivity. The ratio between the number of lesions at 6- and 18-connectivity is similar for both raters, but is clearly an order of magnitude difference between Rater #1 and Rater #2. It is tempting to switch connectivity models, however there is a biological ambiguity when making such a change; do we have a single lesion that can be connected across image grid diagonals or do we have two lesions that are grid diagonal connected because of the underlying imaging resolution? Such issues are only compounded when considering 26-connected lesions. We comment on the meaning and impact of this observation in Sec. 6.1.

Our second key observation concerns small lesions and the errors related to identifying such lesions. The vast majority of expert disagreement is among small lesions (61.34% and 75.06% of respective expert disagreement was for lesions with volume ≤10 mm^3^), which is something that is more readily addressable than the interplay of connectivity and image resolution. We can simply suppress all lesions below a certain threshold and report how the SDI varies at different thresholds. Clearly, there is little or no confidence between the raters for small lesions, so excluding such lesions seems like the most appropriate thing in this situation; particularly if it leads to greater agreement between the expert delineations. The mean inter-rater SDI over the 61 data sets is originally 0.5994; if we zero out lesions below 1 mm^3^ then the mean inter-rater SDI is 0.6007, and if we set a threshold of 10 mm^3^ then the mean inter-rater SDI is 0.6029. These SDI numbers are summarized in Table 3, along with their ranges. The effects the threshold has on the number of detected objects is summarized in Table 4.

**Table 3:** Mean, standard deviation (SD), and range of the SDI overlap between the manual raters at different threshold levels on lesion size.

Threshold	SDI		
	Mean (SD)	Range	95% Confidence Interval
0 mm^3^	0.599 (±0.136)	[0.193, 0.793]	[0.565, 0.634]
1 mm^3^	0.601 (±0.136)	[0.194, 0.798]	[0.566, 0.636]^†^
10 mm^3^	0.603 (±0.137)	[0.194, 0.805]	[0.568, 0.638]^†‡^

The 0 mm^3^ corresponds to the original data. We also show the 95% confidence interval of the mean SDI. Statistical comparisons between the different thresholds was done using the two-sided paired Wilcoxon rank test^141^ with correction for multiple comparisons.

^†^Denotes statistical significance at an *α* level of 0.001 when comparing to the 0 mm^3^ threshold.

^‡^Denotes statistical significance at an *α* level of 0.001 when comparing to the 1 mm^3^ threshold.

**Table 4:** We present the number of lesions (percentage) in each detection class for the three different threshold levels on lesion volume.

Threshold	Detection Classes Lesion Count (Percentage)		
	Expert Agreement	Expert Disagreement	Ambiguous Masks
0 mm^3^	1,796 (15.97%)	8,902 (79.16%)	547 (4.86%)
1 mm^3^	1,792 (24.67%)	5,031 (69.26%)	441 (6.07%)
10 mm^3^	1,433 (33.81%)	2,512 (59.27%)	293 (6.91%)

The 0 mm^3^ threshold corresponds to the original data. See Table 1 for descriptions of the corresponding classes and Fig. 1 for examples.

While Fig. 5 informs us about the object detection agreement between our two raters, Fig. 6 highlights how much agreement there is on a lesion-by-lesion basis. For each category, we show a scatterplot showing per-lesion Dice as a function of lesion volume. Fitted curves represent the average lesion-level SDI values across lesion volumes, estimated using locally estimated scatter-plot smoothing (LOESS)^139,140^. This was computed using the LOESS implementation in R with tricubic weighting. For the Expert Agreement class, we see average rater agreement is very consistent across the range of all lesion volumes, ranging from 0.56 to 0.62. However, this is disappointing as we would expect that when raters agree that a single lesion is present—which they do for the Expert Agreement class—they would have a similar interpretation about the lesion boundary. For context, an SDI of 0.6$\overline{\,6\,}$ means that the raters effectively disagree on 50% of the voxels. For example, if Rater #1 identified one voxel for a lesion and Rater #2 identified two voxels for the same lesion, with the raters having just a single voxel overlap; then the SDI for the lesion would be 0.6$\overline{\,6\,}$. More generally, if Rater #1 identifies a lesion as having $r$ voxels and Rater #2 uniquely identifies the same lesion as having $2r$ voxels with an overlap of $r$ voxels between them then the SDI would remain 0.6$\overline{\,6\,}$. Which highlights the volume insensitivity of SDI and more importantly, the high level of disagreement between the human raters.

### Algorithm comparison

**Figure 7:**
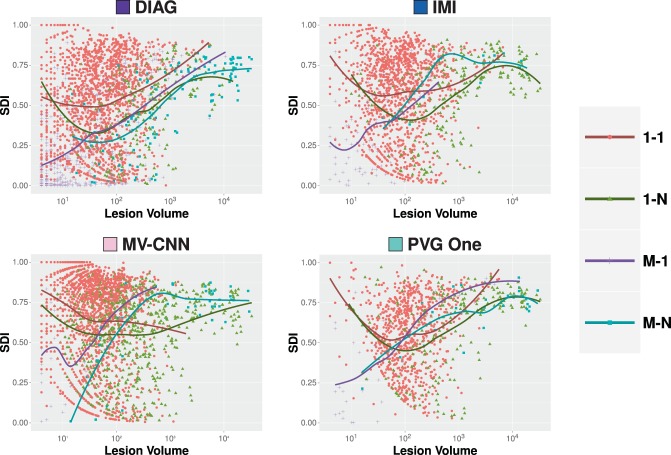
For our four comparison algorithms, we show per-lesion SDI against the Consensus Delineation as a function of the lesion volume (color coded by lesion classification). For each category, the dots are individual lesions and the solid lines are best fits based on LOESS^139,140^.

**Figure 8:**
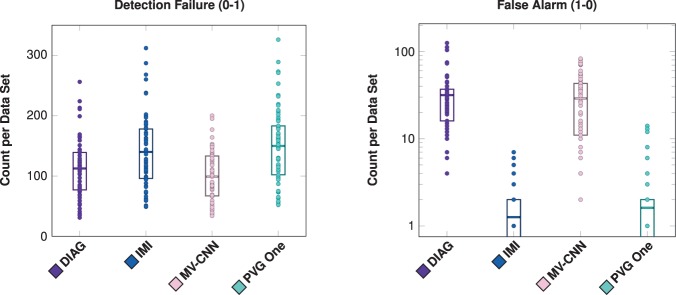
Shown for all four comparison algorithms (DIAG, IMI, MV-CNN, and PVG One) are the number of detection failures and false alarms (shown with a log scale) on a per data set basis. For each plot, a dot denotes the respective count for one of the 61 test data sets, the rectangles represent the inter quartile range (IQR), and the horizontal bars are the means. When the IQR reaches the bottom of the graph it extends to zero.

Next, we compare the four algorithms to the consensus delineation. We show the per-lesion SDI trends for each of the following classes: correct detection (1-1), merge (1-N), split (M-1), and split-merge (M-N). These classes represent the cases of agreement between the various algorithms and the Consensus Delineation, and are shown in Fig. 7. The average lesion-level SDI values across lesion volumes was estimated using LOESS (computation described in Section 4.1). The other two classes (detection failure (0-1) and false alarm (1-0)) do not fit neatly within these plots as they have an SDI of zero and in the latter no true lesion volume. We present plots of the detection failure and false alarms for each algorithm in Fig. 8, by showing the number of counts per volume basis for both of these classes. From Fig. 7, we observe that while these algorithms have a globally similar SDI, they have very different characteristics on a per-lesion basis and within the context of the four classes presented. This point is also evident when examining Fig. 8: both DIAG and MV-CNN have lower detection failure levels but at the expense of dramatically increased false alarm rates (note that the false alarm rates are shown on a log scale).

**Figure 9:**
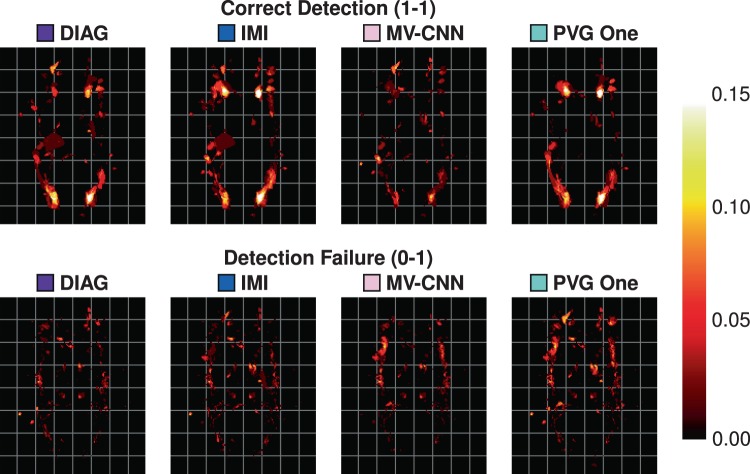
Shown are heat-maps (with grid lines) for the lesions in particular classes. The top row shows the correct detection class for the four comparison algorithms and the bottom row shows the detection failure class.

**Figure 10:**
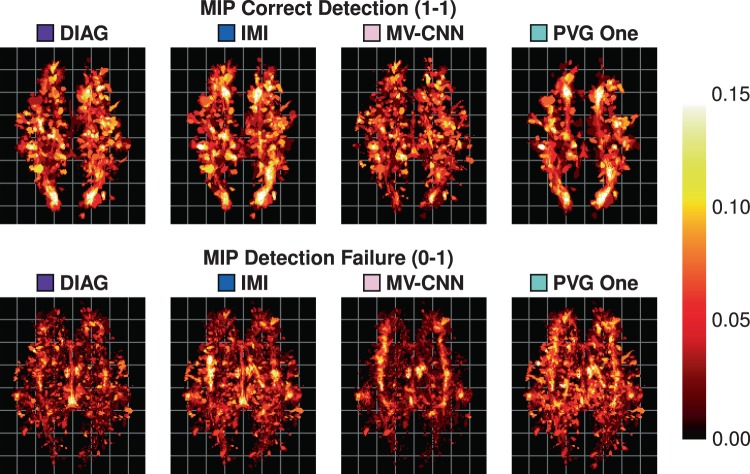
Shown are the axial maximum intensity projections (with grid lines) of the heat-maps for the correct detection class (top row) and the detection failure class (bottom row).

As our data are processed in a common 1 mm^3^ isotropic MNI template, we can create heat maps for each of the different detection classes. We construct these heat maps by taking the class labels for each object and averaging them for each algorithm over the 61 images in our Test data set. A value of 1, at a voxel for a particular class and algorithm, would mean that in all 61 test images the algorithm had lesions of that particular class at that particular voxel with respect to the Consensus Delineation. These heat maps are thresholded at 0.15 to allow better visualization of lower instance values. An example axial slice of these heat maps is shown in Fig. 9, with the corresponding axial maximum intensity projections (MIPs) shown in Fig. 10. Reviewing both Figs. 9 and 10 suggests a very different spatial distribution to the correctly detected lesions. MV-CNN is markedly different from the appearance of PVG One, DIAG, and IMI for the correct detection (1-1) class (shown in Fig. 9); we observe this by noting the distinctive shape that the correct detection class has for each of the four algorithms. This is also reflected in the MIPs in Fig. 10, with MV-CNN clearly having more correct detections within the inter-hemispheric fissure and larger posterior lateral extents. Correspondingly, MV-CNN has lower detection failure rates in the inter-hemispheric fissure in the MIP. We also observe the similarities between IMI and PVG One, both having similar distributions around the ventricles on the single axial slices, with some differences when considering the MIP images.

## Hybrid algorithm

**Figure 11:**
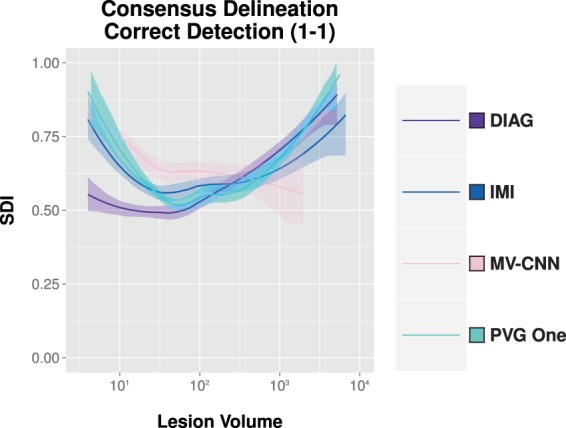
Shown are the regression curves for the correct detection class for each of DIAG, IMI, MV-CNN, and PVG One. Also shown is the 95% confidence band around each regression.

**Figure 12:**
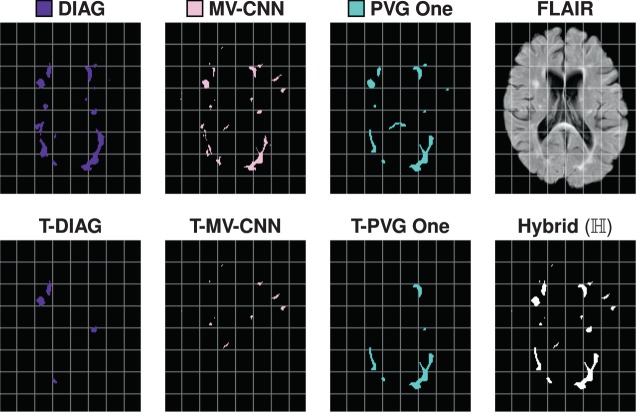
For a test data set, in the top row, we show the axial slices of the DIAG, MV-CNN, and PVG One segmentations, and the corresponding FLAIR image. In the second row, we show the volume thresholded version of DIAG (**T-DIAG**), MV-CNN (**T-MV-CNN**), and PVG One (**T-PVG One**), after the corresponding thresholds have been applied from the 2-Fold variety of our hybrid algorithm. The final image in the second row is the segmentation generated from the union of these results and is denoted **Hybrid** (${\mathbb{H}}$). For this subject, the IMI algorithm did not contribute any lesions and the corresponding images are not displayed.

**Figure 13:**
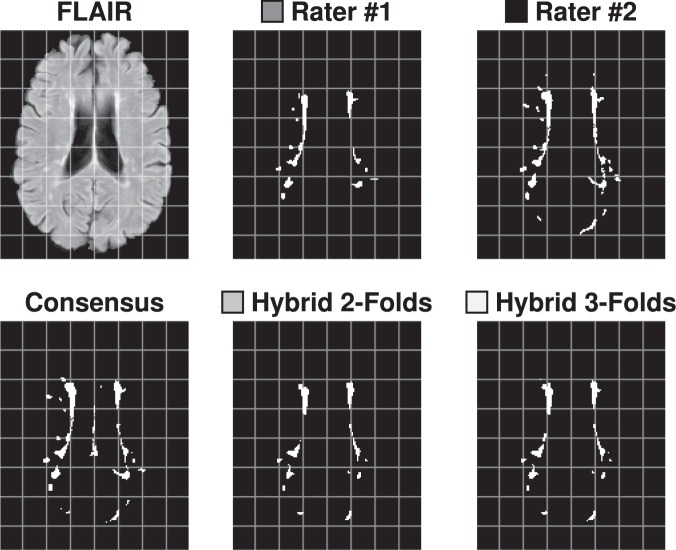
Shown on the top row are an axial slice of the FLAIR image for a subject from the Test data set, and the corresponding segmentations by Rater #1, Rater #2. On the bottom row are the corresponding slices for the Consensus Delineation (labeled Consensus) and the hybrid algorithm with 2-folds (labeled Hybrid 2-Folds) and with 3-folds (labeled Hybrid 3-Folds).

From the top four algorithms presented in Section 4.2, we construct a new hybrid WML segmentation algorithm. The goal here is to demonstrate how our refined SDI analysis can provide opportunities to improve existing algorithms. In Fig. 11, we present the correct detection class for each of the four algorithms under consideration and include 95% confidence bands (computation described in Section 4.1). We leverage these four algorithms’ results and our insights from SDI analysis to produce a better WML segmentation. We construct our algorithm, hereafter referred to as Hybrid, based on a cross-validation and a naive volumetric threshold framework. We do this by subdividing the number of subjects into $n$-folds of data: by subject, we mean all time-points of a single subject. We consider the $(n-1)$-folds of data and identify the best performing algorithm in the correct detection class across the striation of lesion volumes. Specifically, starting at the smallest size lesions we identify the best performing algorithm as the algorithm with the highest SDI in the correct detection class, which we denote as ${{\mathcal{A}}}_{1}$. There is a volumetric threshold, ${t}_{1}$, at which ${{\mathcal{A}}}_{1}$ ceases to be the best performing algorithm and is replaced by ${{\mathcal{A}}}_{2}$; which in turn is replaced ${{\mathcal{A}}}_{3}$ at volumetric threshold, ${t}_{2}$. In this manner, we can learn ${{\mathcal{A}}}_{i}$’s and volumetric thresholds ${t}_{i}$ from the $(n-1)$-folds of data, which we subsequently apply to the ${n}^{{\rm{th}}}$ fold. By “apply” to the ${n}^{{\rm{th}}}$ fold, we mean identify lesions with volumes between the thresholds ${t}_{i}$ and ${t}_{(i+\mathrm{1)}}$ belonging to the best performing algorithm in that range, which would be ${{\mathcal{A}}}_{(i+\mathrm{1)}}$ in this case. Our algorithms output is the union of these lesion segmentations. We can formally write the set of lesions, ${\mathbb{H}}$, identified by our algorithm as,

$${\mathbb{H}}=\mathop{\cup }\limits_{i}\{l\in {{\mathcal{A}}}_{(i+1)}|{t}_{i} < {\rm{vol}}(l)\le {t}_{(i+1)}\,{\rm{and}}\,{\rm{vol}}(l)\,{\rm{is}}\,{\rm{indentified}}\,{\rm{by}}\,{{\mathcal{A}}}_{(i+1)}\},$$

were vol(*l*) denotes the volume of the lesion, $l$, which falls between the desired thresholds and was identified by the best performing algorithm in that range. For completeness, we note that ${t}_{0}=0$ and that $i$ is not bound by the number of algorithms under consideration but rather by the number of times the best algorithm changes across the range of lesion volumes. If one algorithm was considered the best in correct detection across the range of lesion volumes than $i$ would be bounded by one. In Fig. 12, we show how the results of a hybrid algorithm are constructed. In particular, we show the original segmentations, the segmentations after the appropriate volume thresholds have been identified and the corresponding union of those thresholded segmentations. In Fig. 13, we show an example axial slice of a FLAIR image and the corresponding segmentations from both raters, the consensus delineation, and the outputs of using the hybrid algorithm with 2-folds (Hybrid 2-Folds) and with 3-folds (Hybrid 3-Folds).

**Figure 14:**
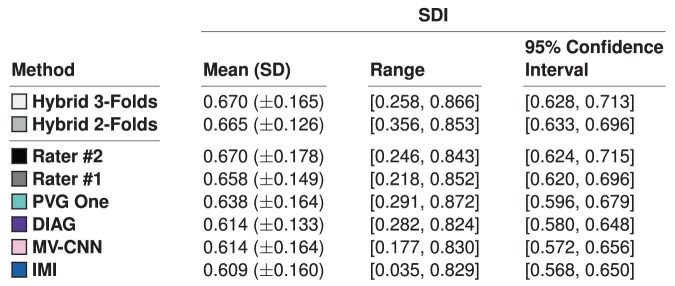
Mean, standard deviation (SD), and range of the SDI overlap scores against the Consensus Delineation for Hybrid, with cross-validation using two- and three-folds are shown in the top two rows. We also show the 95% confidence interval of the mean SDI. Hybrid 2-Folds is based on a two-fold cross validation from the results of DIAG, IMI, MV-CNN, and PVG One; Hybrid 3-Folds is the three-fold cross validation result from the same data. We train on $(n-1)$-folds and test on the *n*-fold; repeating this process by cycling through the various folds, with the combined results presented.

We construct results for our hybrid lesion segmentation algorithm, based on cross-validation using two- and three-folds. To verify the utility of our hybrid lesion segmentation algorithm, we compute the mean SDI against the Consensus Delineation (Fig. 14) and include the results to those for DIAG, IMI, MV-CNN, and PVG One. As we can see from Fig. 14, either one of our cross-validated algorithms has a substantially higher mean SDI than any of the four algorithms against the Consensus Delineation. Hybrid with 2-Folds has a smaller standard deviation and correspondingly tighter 95% confidence interval for its mean SDI, than the other methods. To test the significance of these result we compute a two-sided Wilcoxon Signed-Rank Paired Test^141^ with a correction for multiple comparisons between the two versions of our algorithm and each of the four algorithms under consideration here. Hybrid with 3-Folds is statistically significantly better than all the reported methods at an $\alpha$-level of 0.01, except PVG One. Meanwhile, Hybrid with 2-Folds is not statistically significantly different than any of the reported methods at an $\alpha$-level of 0.01. Importantly, our 2-Fold and 3-Fold hybrid algorithms are the first results on the 2015 Longitudinal Lesion Segmentation Challenge data to match the performance of the manual segmentations against the Consensus Delineation.

## Discussion and Conclusions

The most important aspect of this work is in demonstrating the potential wealth of information that can be gleaned from refined analysis of medical image segmentations. We also showed that simple modifications to rater delineations and algorithms can enhance the desired outcomes. This work is not intended to be a comprehensive review of available segmentation measures or the relative merits of such measures, but rather an exploration of what could be learned from such measures. As noted earlier, we have focused this work on the SDI, however this approach could be applied to any segmentation measure. We make no claim that the SDI is the most appropriate metric for segmentation evaluation; however, given its prevalence it seems prudent to maximize the information that it can provide. Below we review and consider the potential impact of our case studies.

### Inter-rater comparison

For the inter-rater comparison, we could see the raters had a different interpretation of the viable size of lesions that they could identify: Rater #1 had 904 lesions ≤10 mm^3^ whereas Rater #2 had 6800 lesions in the same range. At the time of writing we can not confidently explain this surprising disparity between the raters; it may represent the confidence each rater has in their own ability to identify small lesions or the manner in which the raters used the delineation toolkit. The small lesion size is also related to the choice of connectivity, however changing from 6-connected to 18-connected does not fully mitigate the issue. The SDI within the Expert Agreement class was in the range 0.56 to 0.62, which is disappointing. Unfortunately, the level of disagreement is an area of far greater concern; see Table 4 for example.

These issues are addressable to some extent. We can identify lesions below a certain volume as being unreliable and remove them from the segmentation through thresholding. From Table 3, we see the effect of thresholding is statistically significant. However, the magnitude of the improvement for SDI is minor. Thresholding on lesion size has a more dramatic impact at reducing the number of lesions that are in the Expert Disagreement class, see Table 4. We note that the choice of thresholds for valid manual lesion segmentation in the literature is in a similar range, with Filippi *et al*.^142^ suggesting that a valid lesion must have an extent ≥3 mm in at least one plane and Mike *et al*.^143^ suggesting a 3 mm minimum diameter visible in all three orthogonal views. This is an important point as it reaffirms the correctness of the thresholds outlined by Filippi *et al*.^142^ and Mike *et al*.^143^ We can also enforce connectivity rules when the raters are delineating the lesions. Furthermore, for those lesions in the correct detection class, we can show both rater’s contours of their respective boundaries superimposed on MR images and use this to help them either (1) refine their respective boundaries, (2) reach a consensus, or (3) use the images to help improve future rater training.

Using the Ambiguous Masks class also highlighted some positive aspects of our rater behavior. The low incidence of cases in the split-merge category (see Fig. 5) is encouraging. It suggests that it is rare for raters to identify lesion groups in a dissimilar manner. That is, if one rater sees M lesions in a region our second rater was unlikely to identify N different lesions within the same region; with those M and N lesion being different partitions of the same lesions.

### Algorithm comparison and hybrid algorithms

From Fig. 4, we see that our four comparison algorithms have similar SDI against the Consensus Delineation; however from Figs. 7 and 8 we note that each of these four algorithms exhibits very different performance characteristics within each SDI class. For example, both DIAG and MV-CNN have lower detection failure levels but dramatically higher false alarm rates (see Fig. 8). However, it is these different performance characteristics that allowed us to build our 2-Fold and 3-Fold hybrid algorithm results. Our 2-Fold and 3-Fold were derived solely based on the correct detection (1-1) class behavior of each algorithm (see Fig. 11). Figure 12 shows an example of how lesions of different morphological properties (shape, size, and location) coming from different algorithms can contribute to the results of the a hybrid algorithms. In this particular example, MV-CNN contributed lesions which it identified as *small*; DIAG contributed lesions which it identified as *medium*; and PVG One contributed lesions which it identified as *large*. While IMI did not contribute any results. Figures 12 and 13 demonstrate that a variety of morphological features can be captured by the hybrid style fusion algorithms. However, richer and more diverse forms of fusing–than the proposed straightforward union operation–could provide improved further potential improvements, see discussion below.

Furthermore, we can understand the failure characteristics of each of the algorithms from Fig. 8; in particular, IMI and PVG One have very low false alarm (1-0) rates. We could have used this to help further refine our 2-Fold and 3-Fold results. For example, we could use the lesions identified by either IMI, PVG One, or both to identify lesions while using our volumetric selection scheme to determine the extents of those same lesions in our 2-Fold and 3-Fold final hybrid segmentation. We could additionally incorporate our heat maps to further improve our 2-Fold and 3-Fold results by filtering out regions where an algorithm is prone to produce false-alarms or lowering detection criteria in areas subject to detection failure. These examples illustrate the many different ways in which the presented analysis could be used by algorithm developers to improve their methods.

Our 2-Fold and 3-Fold results were constructed using cross-validation; however, there was knowledge of the correct-detection class performance with the training folds which could be a point of criticism. Ultimately, either of the proposed hybrid style fusion algorithms are bound by the accuracy of the available algorithms which is obviously a limiting factor. The point of this work is not to present a new lesion segmentation algorithm but rather to demonstrate the relative ease with which an algorithm could be formulated by leveraging the proposed advanced evaluation methods. The focus of this paper is not be the proposed algorithms, but the potential use of the new evaluation method to offer insight about the qualities and deficiencies of an algorithm (Sec. 4.2) or for comparison of human raters (Sec. 4.1). However, there are two striking observations, first either version of our hybrid algorithm would have been the top ranked algorithm in the 2015 Longitudinal Lesion Segmentation Challenge (as shown in Table 3 in Carass *et al*.^115^) Second, our 3-Fold version is generating results at a level consistent with the top ranked human rater. Moreover, it is our plan to update the 2015 Longitudinal Lesion Segmentation Challenge Website (http://smart-stats-tools.org/lesion-challenge) to offer plots similar to Fig. 6 for each new participant. It is our hope that these plots will help highlight opportunities for algorithmic improvement for submitted results.

### Future work

We have been focused throughout this work on improving our understanding of segmentations where the number of objects is not known *a priori*. However, it is interesting to consider applications of this work wherein the number of objects is known and the population of images of such objects is large. In such instances it may be difficult to review the individual segmentations to appreciate systematic trends. It is one of our postulates that figures showing SDI vs. object volume would highlight any anomalous behavior, allowing for more rapid review of rater delineations or correction of erroneous algorithm outcomes.

It would be interesting to explore our SDI framework in a location specific manner. According to our heat maps (Figs. 9 and 10) it is feasible to conceive that one algorithm would have superior performance in the frontal temporal lobe, for example, and that the same algorithm may be prone to errors periventricularly. Once such performance characteristics are known, it becomes straightforward to post-process an algorithm’s segmentation by boosting probabilities in high confidence areas or removing objects in error prone areas.

We exclusively used the SDI within the Nascimento nomenclature, however we could have also used other measures to explore the inter-rater behavior or when comparing the four algorithms. In particular, instead of exploring the six different detection classes in terms of lesion SDI and volume, we could have considered a multi-dimensional representation (similar to the work by Commowick *et al*.^118^) including the aforementioned quantities and any number of informative measures; such as the Hausdorff^144^ distance or ventricular or cortical mantle distance. The latter could offer interesting insights into algorithm behavior in the juxtacortical, leukocortical, intracortical, and subpial regions–which is the next frontier in MS lesion segmentation.

Simultaneous with the publication of this paper, we plan to make the code for generating SDI classifications and plots similar to Fig. 6 available from http://iacl.jhu.edu/. The 2015 Longitudinal Lesion Segmentation Challenge Website, currently offers a curt report of the performance of newly submitted results. It is our hope to also update the Website to offer plots similar to Fig. 6 for each new participant.

## Data Availability

The Challenge training and test data is available from http://smart-stats-tools.org/lesion-challenge.
